# A novel dual MoS_2_/FeGA quantum dots endowed injectable hydrogel for efficient photothermal and boosting chemodynamic therapy

**DOI:** 10.3389/fbioe.2022.998571

**Published:** 2022-08-30

**Authors:** Zeming Liu, Ning Zeng, Jing Yu, Chunyu Huang, Qinqin Huang

**Affiliations:** ^1^ Department of Molecular Pathology, The Second Affiliated Hospital of Zhengzhou University, Zhengzhou, China; ^2^ Department of Plastic and Cosmetic Surgery, Tongji Hospital, Tongji Medical College, Huazhong University of Science and Technology, Wuhan, China; ^3^ Department of Radiation and Medical Oncology, Hubei Key Laboratory of Tumor Biological Behaviors, Hubei Cancer Clinical Study Center, Zhongnan Hospital of Wuhan University, Wuhan, China

**Keywords:** hydrogel, fenton reaction, photothemal therapy, quantun dot, chemodynamic therapy

## Abstract

Due to its responsiveness to the tumour microenvironment (TME), chemodynamic therapy (CDT) based on the Fenton reaction to produce cytotoxic reactive oxygen species (ROS) to destroy tumor has drawn more interest. However, the Fenton’s reaction potential for therapeutic use is constrained by its modest efficacy. Here, we develop a novel injectable hydrogel system (FMH) on the basis of FeGA/MoS_2_ dual quantum dots (QDs), which uses near-infrared (NIR) laser in order to trigger the synergistic catalysis and photothermal effect of FeGA/MoS_2_ for improving the efficiency of the Fenton reaction. Mo^4+^ in MoS_2_ QDs can accelerate the conversion of Fe^3+^ to Fe^2+^, thereby promoting the efficiency of Fenton reaction, and benefiting from the synergistically enhanced CDT/PTT, FMH combined with NIR has achieved good anti-tumour effects *in vitro* and *in vivo* experiments. Furthermore, the quantum dots are easily metabolized after treatment because of their ultrasmall size, without causing any side effects. This is the first report to study the co-catalytic effect of MoS_2_ and Fe^3+^ at the quantum dot level, as well as obtain a good PTT/CDT synergy, which have implications for future anticancer research.

## Introduction

Traditional tumour treatment approaches including surgery, chemotherapy and radiation each have their own set of issues such as multidrug resistance, poor patient status after surgery and so on (Chang et al., 2020; Liu et al., 2020; Liu et al., 2021a). The development of nanotechnology has opened up new opportunities for the cancer treatment (Xie et al., 2019; Yang et al., 2020; Almanghadim et al., 2021; Sargazi et al., 2021). Targeting specific tumour microenvironments (weak acidity, hypoxia, high content of hydrogen peroxide (H_2_O_2_)) with diverse nano systems can effectively remove malignancies (Zhu et al., 2022a). The iron-based nanomaterials could react with high levels of H_2_O_2_ in tumors (a typical Fenton reaction) in order to generate reactive oxygen species (ROS) to kill tumors (Lee et al., 2017; Cao et al., 2020; Yang et al., 2021a; Zhu et al., 2022b). For instance, Liu et al. created a BSO/GA−Fe (II)@liposome to increase the intratumoral oxidative stress, and consequently decrease the development of the tumour growth in the treated animals without causing any further negative effects (Dong et al., 2019). Sang and co-workers built a PZIF67-AT-based H_2_O_2_ homeostasis disruptor to realize the accumulation of H_2_O_2_ in tumour sites through promoting H_2_O_2_ generation as well as restraining H_2_O_2_ elimination for intensive chemodynamic therapy (CDT) (Sang et al., 2020). Nevertheless, the effect of single CDT is limited, and the lower conversion efficiency of Fe^3+^ into Fe^2+^ will also inhibit the effect of CDT (Dong et al., 2020).

Near-infrared (NIR) light-based photothermal therapy (PTT) has showed considerable promise as a minimally invasive, precise tumour treatment (Chen et al., 2021a; Qi et al., 2022). For instance, Mxene, gold nanorods, MoS_2_, etc. have been designed to reach the tumour in nanometer size through the enhanced permeability and retention effect (EPR) effect to obtain effective PTT (Fatima et al., 2021). Unfortunately, it has been found that cells exposed to hyperthermia-treated quickly develop a resistance to heat stress, a condition known as thermotolerance, which considerably boosts their survival (Sargazi et al., 2022). It has been suggested that high-power laser therapy or repetitive light irradiation can achieve effective therapeutic efficiency (Chen et al., 2021b; Liu et al., 2021b). Nevertheless, this could result into adverse risks, which includes inflammatory disease and tumour metastasis (Wu et al., 2019). Multimodal therapy has been expected to solve this issue. For instance, MoS_2_ cannot only achieve photothermal effect, but also catalyse Fe-based Fenton reaction to enhance the effect of CDT (Zhang et al., 2020). Nevertheless, the larger size of MoS_2_ nanosheets is not conducive to metabolic clearance and retains potential long-term toxicity (Wang et al., 2016; Li et al., 2019), so this problem must be resolved, and intravenous injection of nanomaterials often faces problems such as uncontrollable early leakage of cargo.

Systemic drug therapy such as intravenous injection is the preferred way for treating many diseases, particularly cancer, is systemic medication therapy (Aliakbar Ahovan et al., 2020; Arshad et al., 2021; Mellati et al., 2021). This treatment modality has been restricted by side effects, off-target accumulation, toxicity, and rapid renal and hepatic clearance (Li et al., 2022; Xue et al., 2022). Instead, a macro therapeutic vehicle that incorporates the medicine can improve retention, reduce metabolic clearance, and lessen side effects (Khosravimelal et al., 2021; Simorgh et al., 2021). Higher effective doses are delivered topically while the therapeutic molecule’s stability is improved, minimizing side effects, clearance, and build-up in the liver and kidneys after systemic delivery (Qiu et al., 2018). Hydrogels have the structure of cross-linked three-dimensional networks and could be utilised as efficient drug carriers, wherein photo responsive hydrogels can be stimulated by light to provide local drug delivery (Zhao et al., 2021). They have demonstrated potential in a variety of therapeutic applications as biocompatible materials (Lai et al., 2020; Lai et al., 2021). For example, Zhu et al. (2021a) designed a PB hydrogel to achieve PTT/PDT. By adjusting the laser power and spot size, this hydrogel can regulate how effectively the carrier releases. This motivates us to load quantum dots into hydrogels to achieve tumour suppressive effect.

**SCHEME 1:**
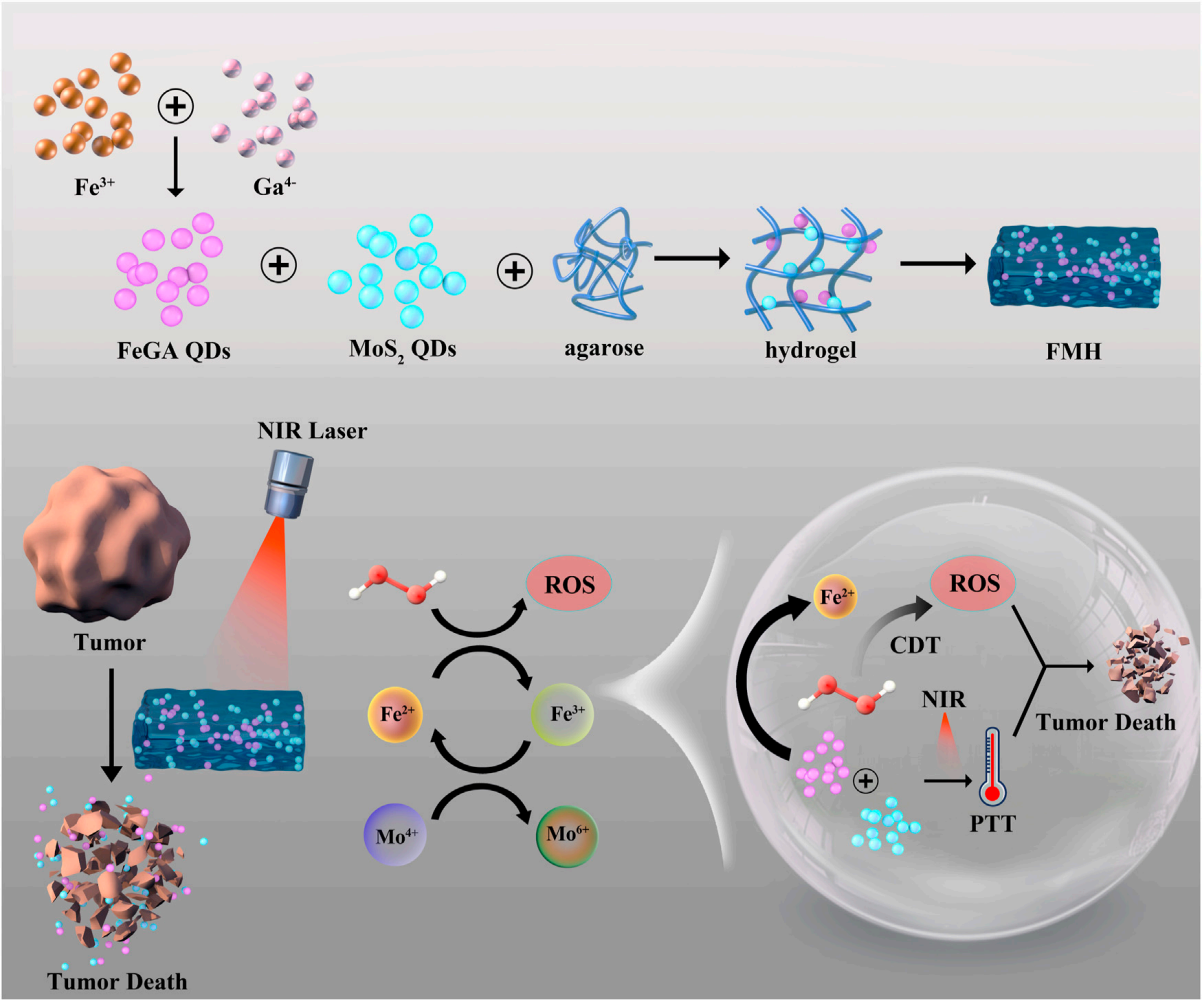
Schematic illustration of a novel dual MoS_2_/FeGA quantum dots endowed injectable hydrogel for efficient photothermal and boosting chemodynamic therapy.

For scaled-up PTT/CDT, we build a dual quantum dot (QD)-based injectable hydrogel technology in this study (Scheme 1). FeGA QDs and MoS_2_ QDs are co-encapsulated into agarose hydrogels to create FMH, which remains at the tumour site following intratumoral injection for a considerable amount of time. Additionally, owing to the broad absorption bands of FeGA and MoS_2_ in the near-infrared region, FMH could respond to 808 nm laser to realize the conversion of light energy to thermal energy, which can make the hydrogel warm and soften, and accelerate the release of MoS_2_ and FeGA while obtaining PTT. Notably, MoS_2_ could be utilised as a co-catalyst to reduce Fe^3+^ to Fe^2+^ with higher Fenton reaction activity, thereby improving the CDT effect. Agarose’s ability to be expelled with metabolism once therapy is over, and the ease with which small-sized quantum dots could be eliminated by metabolism all speak to the safety of the treatment method. This report is the first to verify the co-catalytic effect of MoS_2_ and Fe^3+^ at the quantum dot level, and acquire a good PTT/CDT synergistic anti-tumour, which have implications for future anti-cancer research.

### Preparation of FMH

**FIGURE 1:**
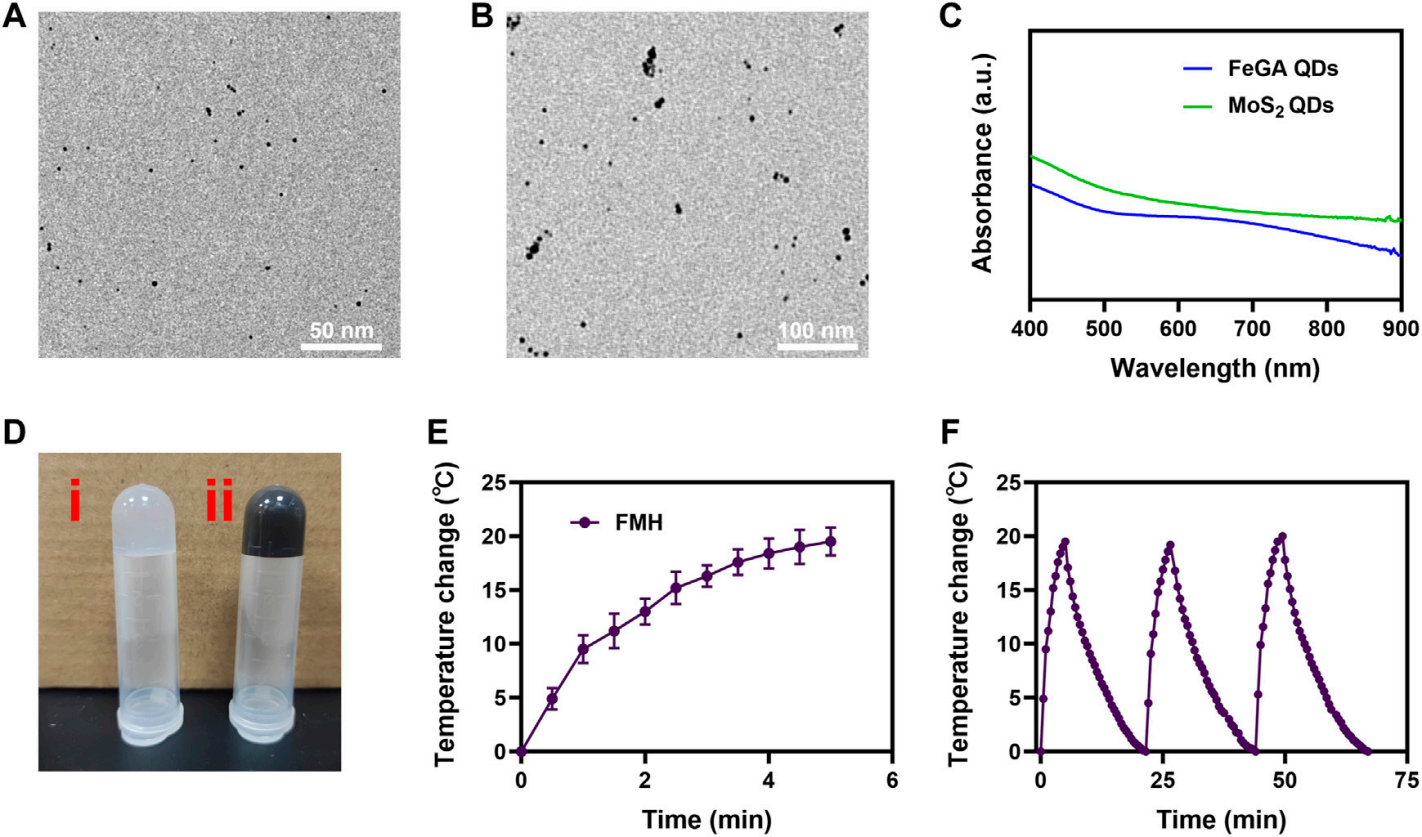
**(A)** TEM image of FeGA QDs. **(B)** TEM image of MoS_2_ QDs. **(C)** UV-vis-NIR spectra of FeGA QDs and MoS_2_ QDs. **(D)** Photographs of (i) pure hydrogel and (ii) FMH. **(E)** The temperature profile of FMH irradiated by NIR laser (808 nm, 0.5 W/cm2), followed by natural cooling to room temperature. **(F)** The photothermal stability (three laser on/off cycles) of FMH.

FeCl_3_ were added to gallate solution to create FeGA quantum dots (Yang et al., 2021b), which were then analysed using transmission electron microscopy (TEM), the result showed that the diameter of FeGA we prepared is about 2–4 nm (Figure 1A). And Figure 1B showed that the size of MoS_2_ quantum dots was approximately 10 nm. In contrast to the conventional nanomaterials (100–400 nm), quantum dots (QDs) have better metabolic clearance, which is more conducive to clinical performance (Wang et al., 2022). Additionally, the UV-Vis absorption spectra (Figure 1C) demonstrated that FeGA and MoS_2_ exhibits strong NIR region absorption. We then created a composite FMH system by adding FeGA QDs and MoS_2_ QDs to agarose hydrogels (Figure 1D) (1) is pure agarose hydrogel, 2) is FMH), the hydrogel would slowly solidify at room temperature, and the hydrogel placed upside down will not flow down the tube wall. Results from scanning electron microscopy (SEM) showed that FMH had a pore structure (Supplementary Figure S1), which is beneficial for the loading and releasing of cargo from the hydrogel.

### Verifying the photothermal stability of FMH

An external light stimulus can cause changes in the light-responsive hydrogel. We utilised an 808 nm laser to verify the heating performance of the FMH. As demonstrated in Figure 1E, owing to the simultaneous loading of FeGA and MoS_2_, the FMH could be subjected to lower power near-infrared. A better heating effect was made possible by the irradiation, and the FMH system’s temperature increased by about 19.2°C in 5 min. Then, the photothermal stability of FMH was measured under 808 nm laser irradiation (laser turned on for 5 min), and then cooled (laser turned off) to naturally return to the initial temperature for 3 successive cycles (Figure 1F). According to the results, the heating characteristics of FMH failed to show obvious attenuation after 3 irradiations, which indicated that FMH is a reusable durable photothermal agent for PTT.

### The structure of FMH

**FIGURE 2:**
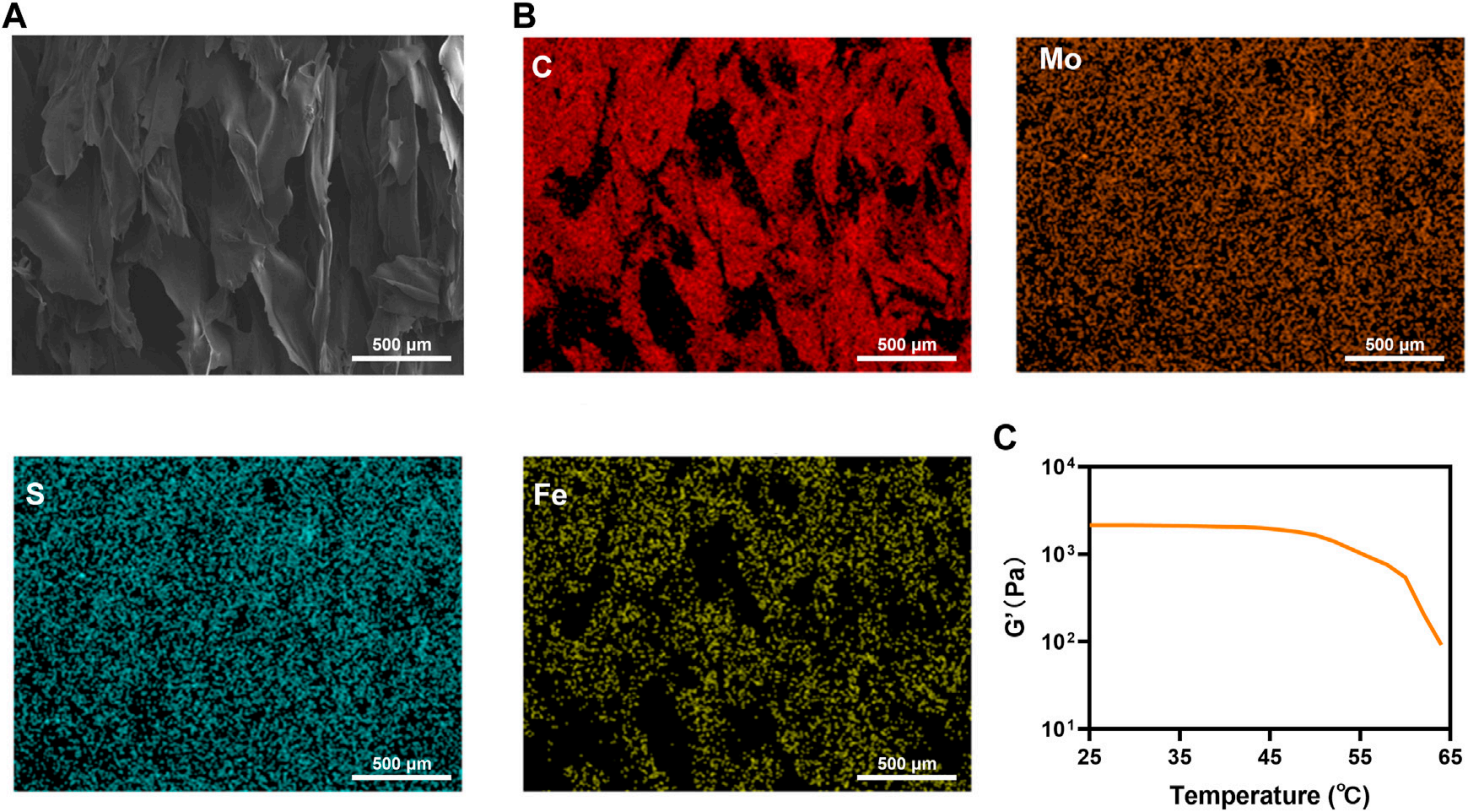
**(A)** SEM of FMH hydrogel; **(B)** Elemental distribution mapping of FMH hydrogel: C; Mo; S and Fe. **(C)** Rheological curves of FMH.

Figure 2 A also depicts the hydrogel’s rough structure. After the FMH freeze-drying treatment, the EDS analysis was carried out. The results demonstrated that the hydrogel contained a large amount of C, Mo, S, and Fe elements (Supplementary Figure S2), and these elements were distributed in the hydrogel uniform, which indicated that the FeGA and MoS_2_ QDs are homogeneously mixed with the hydrogel (Figures 2A,B). The rheological characteristics of FMH were further confirmed. The storage modulus of FMH continued to decrease with the increase of temperature, which had been beneficial to the release of quantum dots.

### Evaluating the photothermal effect of FMH

**FIGURE 3:**
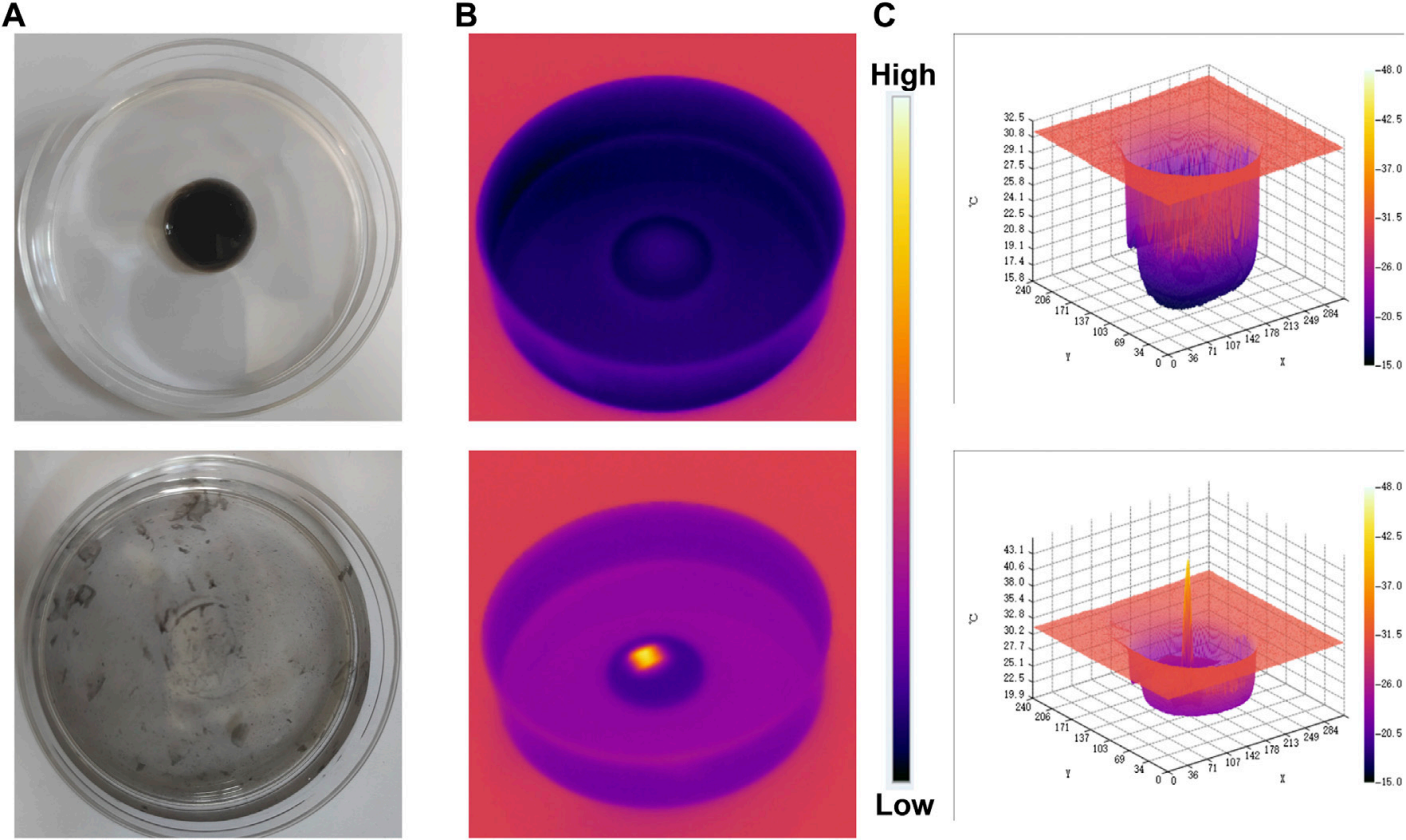
**(A)** The morphology of the prepared FMH before and after 0.5 W/cm2 808 nm laser irradiation for 5 min. **(B)** The infrared thermal images of the prepared FMH before and following irradiation. **(C)** Relevant 3D temperature diagram in 2B.

The excellent photothermal effect of FMH was further propelled by using an experimental setup that looked at FMH’s release behaviour when exposed to near-infrared light (Figure 3B, schematic diagram). Once the hydrogel has solidified in the middle of the glass dish, it was irradiated with an 808 nm laser device. Due to the better photothermal conversion properties of FMH, the temperature of the hydrogel gradually enhances, and the matrix confined in it would gradually flow out with the softening of the hydrogel, which indicated that the FMH could respond well to the laser for releasing the quantum dot material (Figure 3A). Infrared images also further verified that FMH can convert light energy into heat energy (Figure 3C). Thus, it is anticipated that the subsequent biological therapy would be realised. In the presence of Fe^3+^ and H^+^, H_2_O_2_ could be decomposed and converted into highly active hydroxyl radicals, however, the spontaneous process of the reaction between endogenous Fe^3+^ and H_2_O_2_ is within the threshold of cellular redox homeostasis and cannot exert a therapeutic effect. In order to ensure the therapeutic effect (Luo et al., 2016; Min et al., 2020; Bai et al., 2022), it is urgent to inject exogenous Fenton’s reagent to encourage excess hydroxyl radical formation in cancer cells. Thus, loading FeGA into hydrogels is expected to overcome this issue.

### *In vitro* anticancer effect of FMH

**FIGURE 4:**
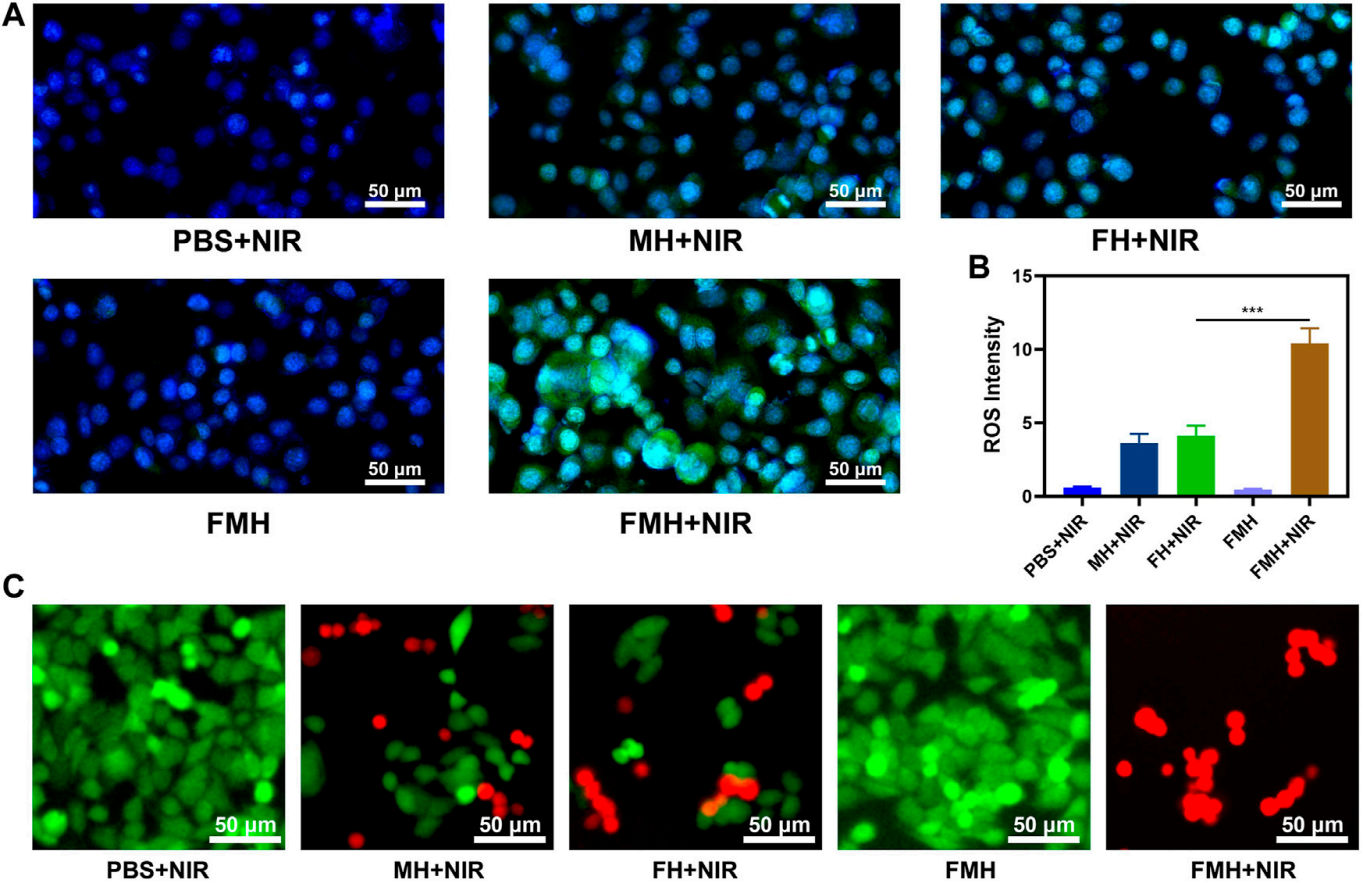
**(A)** Confocal laser scanning microscopy images of ROS generated in 4T1 cells after different treatments. **(B)** Quantitative analysis of ROS production in **(A)**. **(C)** Fluorescence images of 4T1 cells stained with fluorescein diacetate (FDA; live cells, green fluorescence) and propidium iodide (PI; dead cells, red fluorescence) after incubation with different formulations. ***p < 0.005; Student’s t-test.

We separately prepared hydrogels containing only FeGA QDs (FH) and hydrogels containing only MoS_2_ QDs (MH), intracellular reactive oxygen species (ROS) production had been detected using 2′, 7′-dichlorodihydrofluorescein diacetate (DCFH-DA), which could be oxidized to 2′, 7′-dichlorofluorescein (DCF) with green fluorescence in the presence of ROS. The PBS + NIR group exhibited very little of the typical green fluorescence of reactive oxygen species, as seen in Figure 4A. The FMH group without NIR treatment also produced almost no ROS, which indicated that the hydrogels bound the dual quantum dots well. Both the FH + NIR group and MH + NIR exhibited moderate-intensity green fluorescence, which was because of the release of QDs in the corresponding hydrogels after NIR irradiation, then QDs reacted with the high content of H_2_O_2_ in the tumour to generate ROS. Notably, green fluorescence was markedly increased in FMH + NIR, notably greater than in FH and MH alone and significantly higher than in the total of the two. This might be due to the fact that the active Mo^4+^ ions on the surface of MoS_2_ QDs could reduce Fe^3+^ ions to Fe^2+^ ions after the 808 nm laser triggers the release of FeGA and MoS_2_ QDs, accelerating the conversion of Fe^3+^ to active Fe^2+^. Similar findings emerged from quantitative study of ROS production in each group (Figure 4B). This 1 + 1>2 synergistic catalytic strategy is expected to enhance the efficiency of CDT and acquire better tumour cell killing effect. The major cause of oxidative stress in cells is an increase in reactive oxygen species (ROS), which may act on biological macromolecules, causing DNA damage and ultimately cell apoptosis (Zhu et al., 2021b; Jiang et al., 2022). The effect of FMH composites on 4T1 cells was further confirmed using FDA and PI staining, as demonstrated in Figure 4C, the PBS + NIR and FMH groups show the strong green fluorescence [FDA, live cells) and ignorable red fluorescence (PI, dead cells)], the FH/MH + NIR groups all showed an appropriate intensity of tumour cell killing effect. While nearly 85% of 4T1 cells were killed by FMH + NIR-induced PTT/enhanced CDT, this finding suggests that the greatest killing of tumour cells was caused by the synergistic interaction between high levels of ROS and FMH-mediated hyperthermia. Likewise, we measured cell viability in each group using the MTT assay kit, and the results were consistent with the previous results (Supplementary Figure S3). To sum it up, the *in vitro* cytotoxicity experiments showed that our prepared FMH has good potential in CDT/PTT.

### *In vivo* anticancer effect of FMH

**FIGURE 5:**
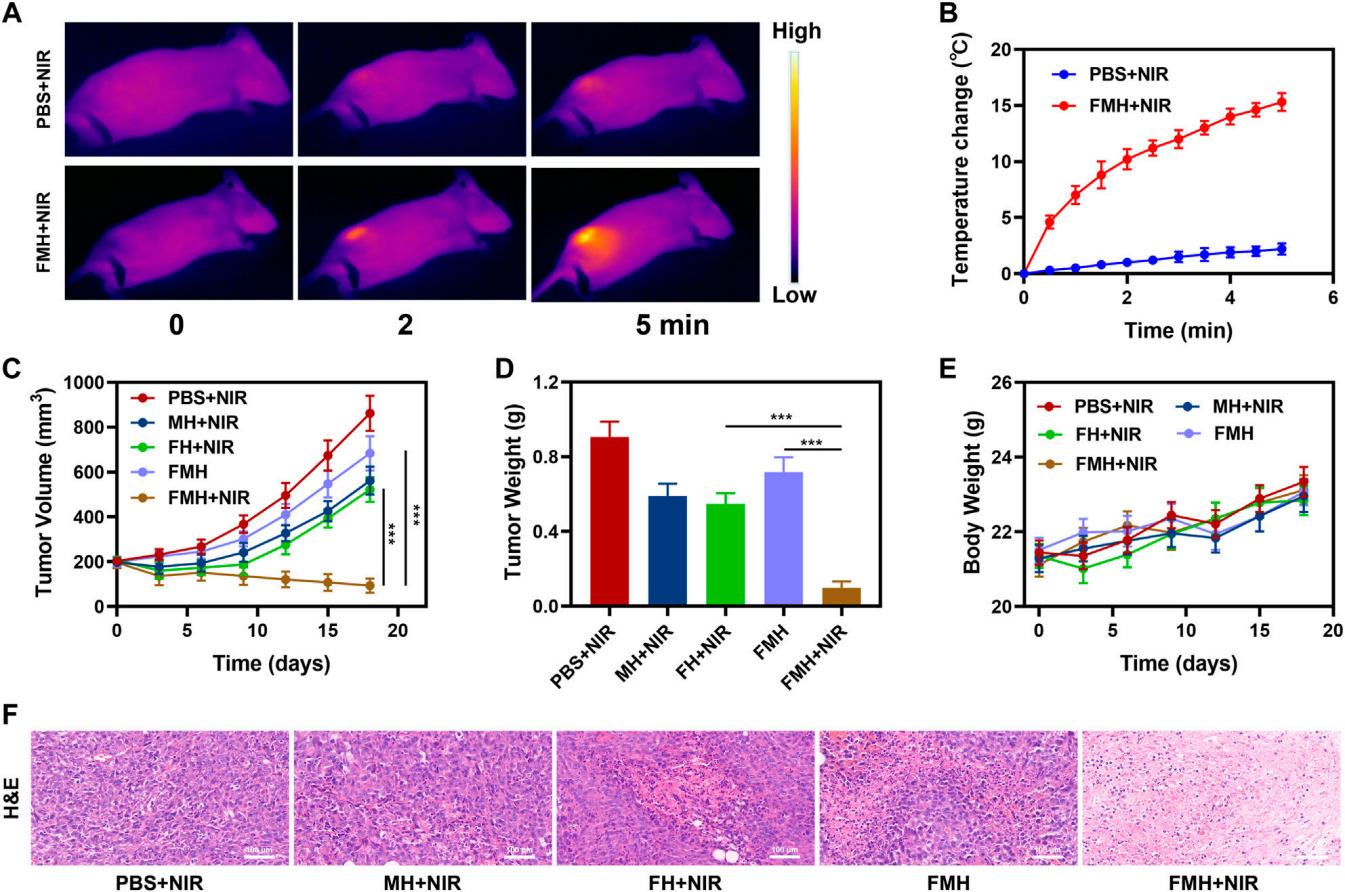
**(A)** Infrared images of the mice tissue under laser irradiation after the indicated treatments. **(B)** Temperature of the mice upon laser irradiation. **(C)** Evolution of the volume of FMH tumors bearing female BALB/C mice after various treatments. **(D)** Evolution of the tumor weight during therapy. **(E)** Body weight of mice recorded every other day for various treatments. **(F)** Relative H&E staining analyses of tumor tissues treated with various treatments. ***p < 0.005; Student’s t-test.

We proceeded to investigate FMH’s *in vivo* anticancer potential in light of the effective *in vitro* antitumor impact that was mediated by FMH. The *in vivo* photothermal properties of the FMH composites were characterized utilizing an infrared thermal imaging camera. As illustrated in Figure 5A, the tumour temperature of mice in the PBS + NIR group barely increased after 5 min of irradiation, while the temperature of the FMH + NIR group rapidly increased about 15.3°C following continuous illumination with a low-power density (0.5 W/cm^2^) 808 nm laser device (Figure 5B), and FMH could acted as a photothermal agent with good response to NIR light, it could acquire efficient PTT and improve the efficiency of Fenton reaction. Because of the non-specific nature of Fe^2+^ in blood circulation, it is counterproductive to directly introduce photothermal agents containing Fe^2+^ into biological systems to achieve the desired therapeutic effect (Dai et al., 2021; Qindeel et al., 2021). These reactive ions may cause severe oxidative damage in normal tissues, possibly resulting into subsequent physiological toxicity, which would further hinder the development of Fenton reaction-based CDT. In the light of aforementioned findings, we continued to explore FHM-based anti-tumor effects *in vivo*. In this study, 4T1 cells had been injected subcutaneously into the mice to establish a 4T1 breast cancer subcutaneous tumour model. When tumours grew to a size of around 200 mm^3^, BALB/c mice with tumours were divided into five groups at random: 1) PBS + NIR; 2) FH + NIR; 3) MH + NIR; 4) FMH and 5) FMH + NIR. Each group was given corresponding treatment. Groups 2, 4 and 5 were administered a 5 mg/kg dose of FeGA QDs. Digital callipers were used to measure the tumour volume every 3 days, and then the weight of the tumour was computed. The experimental results showed that the tumour volume was moderately suppressed in the FH + NIR and MH + NIR groups relative to the PBS + NIR group during the continuous 2-week treatment period, which might be because of the fact that FeGA/MoS alone could only induce lower generation of reactive oxygen species and low PTT (Figures 5C,D). While the tumour volume in the FMH group was only slightly suppressed, it is noteworthy that in the final treatment group, the tumour volume in the FMH + NIR group had been significantly decreased, and the tumour growth was completely inhibited, which benefited from the synergistic photothermal effect of FeGA and MoS_2_, furthermore, MoS_2_ acted as a co-catalyst to accelerate the conversion of Fe^3+^ to Fe^2+^, making the CDT effect better. The findings of H&E staining revealed that the FMH + NIR-induced treatment group had blatant tumour cells necrosis, with cell shrinkage and nuclear compression (Figure 5D). Nevertheless, the H&E staining results of normal major tissues and organs did not change significantly. The endocardium, myocardium and epicardium of cardiac tissue were clearly structured. The spleen tissue capsule is composed of dense connective tissue rich in elastic fibers and smooth muscle fibers with uniform thickness, and there is no obvious abnormality. Similar to this, the weight of mice in each group increased steadily during the treatment period (Figures 5C; Supplementary Figure S4), which proved that the FMH-based synergistic treatment method has no obvious damage to normal tissues and organs. In short, the rise of various emerging technologies has brought new hope for various diseases (Zhang et al., 2022), and we will continue to develop new nanomaterials and technological applications to achieve better value in the future.

## Conclusion

To sum it up, we built an injectable hydrogel based on FeGA and MoS_2_ dual quantum dots and characterized their physicochemical and antitumor properties *in vitro* and *in vivo*. FMH exhibits good near-infrared photothermal heating properties and photothermal stability. *In vitro* cell experiments showed that FMH combined with NIR induced high production of ROS and significantly inhibited cell viability. *In vivo* animal experiments show that the system prepared by us can well inhibit the growth of tumor volume during the treatment period, and has almost no toxic side effects. New information on the creation of effective Fenton nano-adjuvants is provided by this synergistic approach based on synergistic catalysis and the photothermal effect produced by NIR light. This system is also plagued by certain problems, and the preparation methods of quantum dots need to be further improved. In the future, we will further design simpler methods and materials to achieve various biological applications.

## Data Availability

The original contributions presented in the study are included in the article/Supplementary Material, further inquiries can be directed to the corresponding author.
