# Supplementary material for: A novel dual MoS2/FeGA quantum dots endowed injectable hydrogel for efficient photothermal and boosting chemodynamic therapy
Source: Front Bioeng Biotechnol. 2022 Aug 30;10:998571. doi: 10.3389/fbioe.2022.998571 (PMC9468328; doi:10.3389/fbioe.2022.998571)
Supplement: Supplementary file 1 [file DataSheet1.docx]

**Experimental Procedures**

**Materials and reagents.**

Iron chloride hexahydrate (FeCl_3_·6H_2_O) and polyvinylpyrrolidone K30 (PVP) were purchased from Sinopharm Chemical Reagent Co., Ltd. The MoS_2_ QDs were provided by Nanjing XFNANO Materials Tech Co., Ltd (Nanjing, China). Agarose was purchased from Yare Shanghai. Reactive Oxygen Species Assay Kit, and MTT Cell Proliferation Assay Kit were obtained from Yeasen Biotech Co., Ltd (China). The other reagents used in this work were purchased from Sinopharm Chemical Reagent (China) and Aladdin-Reagent (China).

**Preparation and characterization of FeGA QDs and FMH**

Fresh GA–Fe was prepared according to the literature[1, 2]. FeCl_3_·6H2O was added into the gallate solution to keep a 1:1 stoichiometry of Fe^3+^ and GA^4−^ for reaction with magnetic stirring and nitrogen supplementation for 1 h, after which the suspension was centrifuged and the precipitate was washed, collected, and stored for further use. The morphology structures of FeGA were observed by the TEM (JEOL-2100). UV-vis spectra of different samples were recorded by the UV-vis spectrophotometry Lambda 35 (Perkin-Elmer).

The general protocol for the hydrogel preparation is as follows. The prepared FeGA (1 mg/mL in PBS) and the MoS_2_ (1 mg/mL in PBS) were mixed into 1% agarose solution to form FMH. Wherein the concentration of FeGA and MoS_2_ was 100 μg/mL, respectively. Photothermal heating curves of FMH under an 808 nm laser irradiation was monitored using an infrared camera (Fotric 225).

**Samples characterization**

Transmission electron microscopy (TEM, LIBRA 200 CS, Carl Zeiss Co., Germany) was measured to characterize the morphology of QDs. UV-vis spectra of different samples were recorded by the UV-vis spectrophotometry Lambda 35 (Perkin-Elmer). Scanning electron microscopy (SEM) images were captured on a Hitachi FE-SEM S4800 instrument with an acceleration voltage of 3 kV. To study the distribution of all elements, elemental mapping and energy-dispersive spectroscopy (EDS, FEI Magellan 400) were performed.

**Cell line.**

4T1 mouse breast cancer cell line was obtained from the Cell Bank of the Chinese Academy of Sciences and incubated in RPMI-1640 medium supplemented with 10% FBS in a humidified atmosphere at 37 °C.

**Animal tumor models**

Female BALB/c mice aged 4-5 week were purchased from Vital River Company (Beijing, China). 100 μL of 4T1 cell suspension (1×10^6^ cells per mL) were subcutaneous injected into each mouse to establish the tumor models. The animal experiments were carried out according to the protocol approved by the Ministry of Health in People’s Republic of PR China and were approved by the Administrative Committee on Animal Research of the second clinical Medicine College of Wuhan University.

**Rheological Test**

Rheology experiments were performed on an Anton Paar rheometer. Hydrogel samples of different temperatures were prepared and gently placed on the middle of a 15 mm diameter parallel plate with a proper gap. Dynamic oscillatory frequency sweep measurements were conducted at a 1% strain amplitude. To prevent the evaporation of water, a lid was prepared on the top.

**Photothermal Conversion Ability of FMH**

An 808 nm NIR laser (Changchun New Industries Tech.Co., Ltd., Changchun, China) with irradiation powers was used to stimulate the concentrations of FMH (MoS_2_ concentration: 100 μg/mL) in an aqueous medium. The photothermal images of the FMH-based suspensions during laser irradiation were recorded every 30 s using an infrared thermal imaging system. The NIR laser source was equipped with a 4 mm diameter laser module with an adjustable power.

**Intracellular reactive oxygen species (ROS) generation**

For determination of ROS levels via fluorescent imaging, 4T1 cells were incubated for 2 h with 5 different groups: (1) PBS + NIR (0.5 W/cm^2^, 5min), (2) MH + NIR, (3) FH + NIR, (4) FMH and (5) FMH + NIR. The MoS_2_ concentration was 100 μg/mL in group 2, 4 and 5. In order to facilitate the study of ROS production, all samples were added directly into the cells in PBS solution. Then, the fluorescent dye, DCFH-DA (10 μmol/L), was added and coincubated for 20 min at 37 °C. ROS level was determined by a confocal laser scanning microscope (CLSM; IX81, Olympus, Japan). The fluorescent intensity of each group was calculated by ImageJ software.

**In vitro anti-cancer effect of FMH**

The in vitro anti-tumor effect was measured by MTT assay. 4T1 cells were seeded in 96- well plates at a density of 5 × 10^3^ cells per well and incubated for 12 h. Afterwards, cells were incubated for 5 different groups: (1) PBS + NIR (0.5 W/cm^2^, 5min), (2) MH + NIR, (3) FH + NIR, (4) FMH and (5) FMH + NIR. Then, 4T1 cells in group 1, 2, 3 and 5 were exposed to 808 nm laser radiation (0.5 W/cm^2^) for 5 min. At the end of the incubation, 5 mg/mL MTT PBS solution was added, and the plate was incubated for another 4 h. Finally, the absorbance values of the cells were determined by using a microplate reader (Emax Precision, USA) at 570 nm. The background absorbance of the well plate was measured and subtracted. The cytotoxicity was calculated by dividing the optical density (OD) values of treated groups (T) by the OD values of the control (C) (T/C × 100%).

To further visualize the cell phototoxicity of each group, 4T1 cells were incubated for 24 h with 5 different groups: (1) PBS + NIR (0.5 W/cm^2^, 5min), (2) MH + NIR, (3) FH + NIR, (4) FMH and (5) FMH + NIR. Then, cells in group 1, 2, 3 and 4 were exposed to 808 nm laser radiation (0.5 W/cm^2^) for 5 min. Then, all cells were washed with PBS, treated with FDA and PI according to the manufacturer’s protocol, and detected under a fluorescent microscope (IX81, Olympus, Japan).

***In vivo* infrared thermography**

To monitor the in vivo photothermal effect, FMH (MoS_2_: 2 mg/kg) was intratumorally injected into the 4T1 cells tumor-bearing mice, and then the tumors suffered from 0.5 W/cm^2^ irradiation for 5 min at 1 h post-injection. PBS injection used as control group. Meanwhile, the temperature at the tumor was monitored using an infrared camera (Fotric 225).

***In vivo* antitumor study**

The mice were firstly divided randomly into 5 groups (each group included 5 mice): (1) PBS + NIR (0.5 W/cm^2^, 5 min), (2) MH + NIR, (3) FH + NIR, (4) FMH and (5) FMH + NIR. Among them, the dose of MoS_2_ in groups 2, 4 and 5 are 2 mg/kg. NIR was conducted 1h after the injection. Mice body weight and tumor volume in all groups were monitored every 3 days. A caliper was employed to measure the tumor length and tumor width and the tumor volume was calculated according to following formula. Tumor volume = tumor length × tumor width^2^ / 2. After 18 days treatment, mice were sacrificed. Five main organs (heart, liver, spleen, lung and kidney) of all mice were harvested, washed with PBS, and fixed with paraformaldehyde for histology analysis. And the tumor tissues were weighed, and fixed in 4% neutral buffered formalin, processed routinely into paraffin, and sectioned at 4 μm. Then the sections were stained with hematoxylin and eosin (H&E) finally examined by using an optical microscope (BX51, Olympus, Japan).

**Statistical analysis**

Data analyses were conducted using the GraphPad Prism 5.0 software. Significance between every two groups was calculated by the Student’s t-test. *P < 0.05, **P < 0.01, ***P < 0.005.


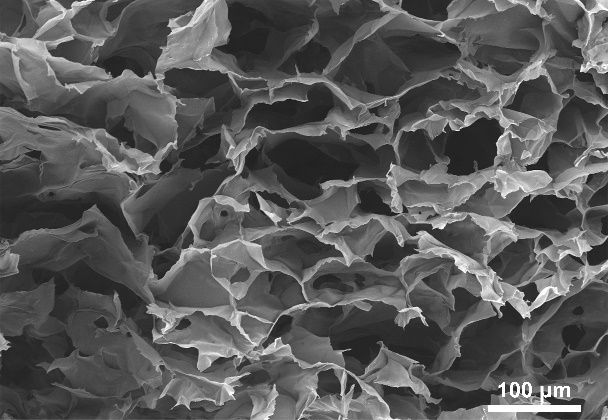


**Figure S1.** SEM image of FMH.


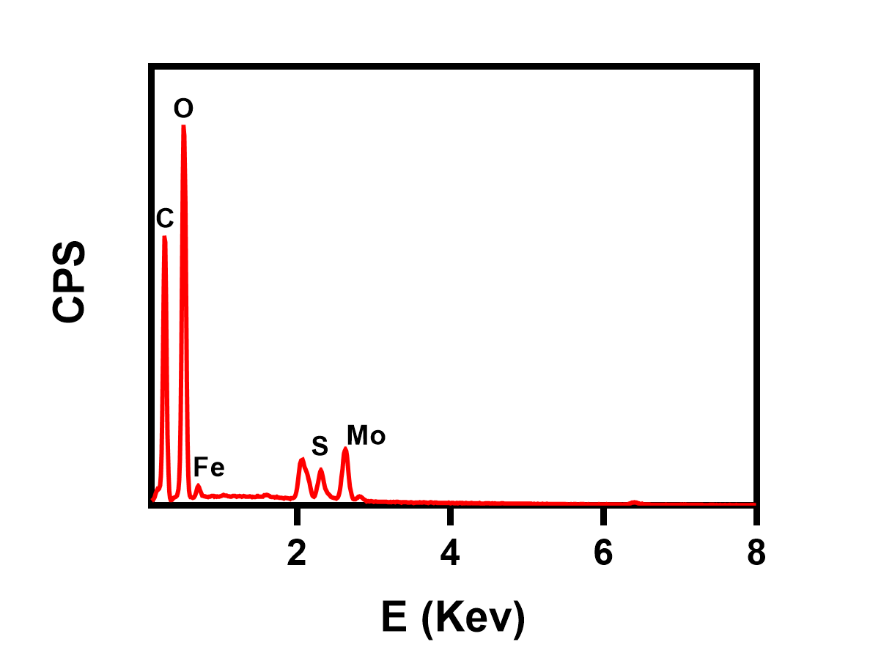


**Figure S2.** EDS of FMH hydrogel.


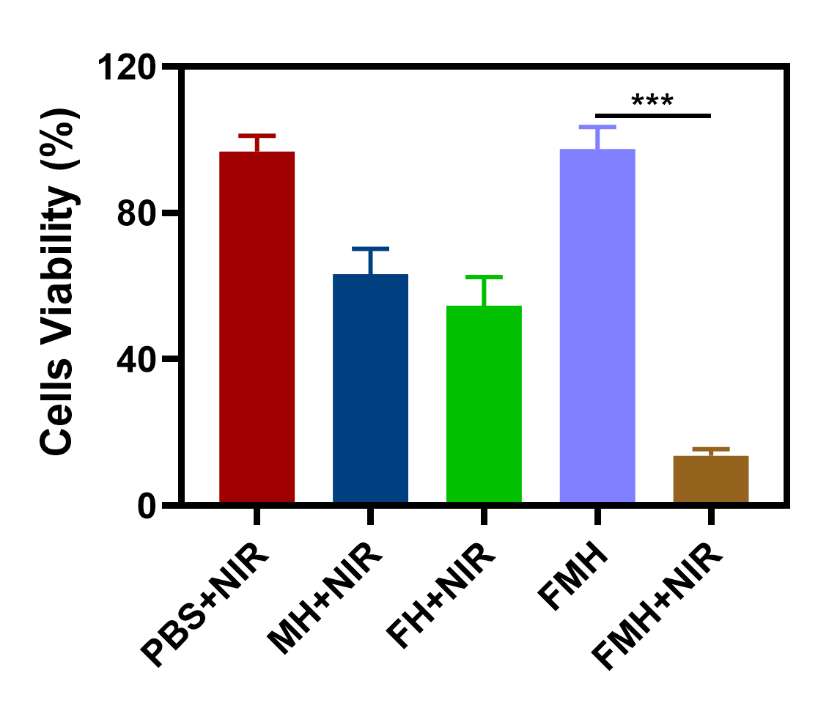


**Figure S3.** Cell viability of 4T1 cells treated with different formulations.


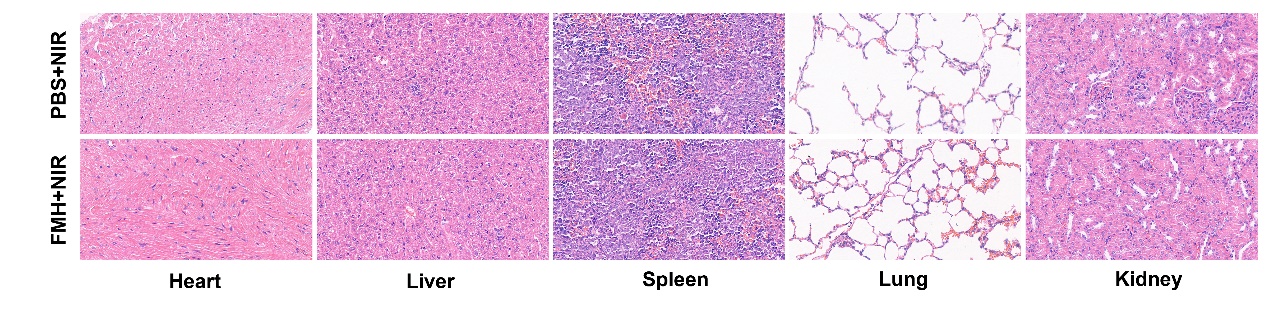


**Figure S4.** Histological data obtained in the major organs (heart, liver, spleen, lung, and kidney) of the mice 14 days after injection under various conditions.

[1] B. Yang, H. Yao, H. Tian, Z. Yu, Y. Guo, Y. Wang, J. Yang, C. Chen, J. Shi, Intratumoral synthesis of nano-metalchelate for tumor catalytic therapy by ligand field-enhanced coordination, Nature communications 12(1) (2021) 3393.

[2] Z. Dong, Y. Hao, Q. Li, Z. Yang, Y. Zhu, Z. Liu, L. Feng, Metal-polyphenol-network coated CaCO3 as pH-responsive nanocarriers to enable effective intratumoral penetration and reversal of multidrug resistance for augmented cancer treatments, Nano Research 13(11) (2020) 3057-3067.
